# Cracking the “Sugar Code”: A Snapshot of *N*- and *O*-Glycosylation Pathways and Functions in Plants Cells

**DOI:** 10.3389/fpls.2021.640919

**Published:** 2021-02-19

**Authors:** Richard Strasser, Georg Seifert, Monika S. Doblin, Kim L. Johnson, Colin Ruprecht, Fabian Pfrengle, Antony Bacic, José M. Estevez

**Affiliations:** ^1^Department of Applied Genetics and Cell Biology, University of Natural Resources and Life Sciences, Vienna, Austria; ^2^La Trobe Institute for Agriculture & Food, Department of Animal, Plant & Soil Sciences, La Trobe University, Bundoora, VIC, Australia; ^3^The Sino-Australia Plant Cell Wall Research Centre, Zhejiang Agriculture & Forestry University, Hangzhou, China; ^4^Department of Chemistry, University of Natural Resources and Life Sciences, Vienna, Austria; ^5^Fundación Instituto Leloir and Instituto de Investigaciones Bioquímicas de Buenos Aires (IIBBA-CONICET), Buenos Aires, Argentina; ^6^Centro de Biotecnología Vegetal, Facultad de Ciencias de la Vida, Universidad Andres Bello, Santiago, Chile; ^7^Millennium Institute for Integrative Biology (iBio), Santiago, Chile

**Keywords:** *Arabidopsis*, glycosyltransferases, plant protein glycosylation, glycan arrays, *O*-glycosylation, *N*-glycosylation, glycosyl hydrolases, glycan functions

## Abstract

Glycosylation is a fundamental co-translational and/or post-translational modification process where an attachment of sugars onto either proteins or lipids can alter their biological function, subcellular location and modulate the development and physiology of an organism. Glycosylation is not a template driven process and as such produces a vastly larger array of glycan structures through combinatorial use of enzymes and of repeated common scaffolds and as a consequence it provides a huge expansion of both the proteome and lipidome. While the essential role of *N*- and *O*-glycan modifications on mammalian glycoproteins is already well documented, we are just starting to decode their biological functions in plants. Although significant advances have been made in plant glycobiology in the last decades, there are still key challenges impeding progress in the field and, as such, holistic modern high throughput approaches may help to address these conceptual gaps. In this snapshot, we present an update of the most common *O*- and *N*-glycan structures present on plant glycoproteins as well as (1) the plant glycosyltransferases (GTs) and glycosyl hydrolases (GHs) responsible for their biosynthesis; (2) a summary of microorganism-derived GHs characterized to cleave specific glycosidic linkages; (3) a summary of the available tools ranging from monoclonal antibodies (mAbs), lectins to chemical probes for the detection of specific sugar moieties within these complex macromolecules; (4) selected examples of *N*- and *O*-glycoproteins as well as in their related GTs to illustrate the complexity on their mode of action in plant cell growth and stress responses processes, and finally (5) we present the carbohydrate microarray approach that could revolutionize the way in which unknown plant GTs and GHs are identified and their specificities characterized.

## Introduction

In the model plant *Arabidopsis thaliana*, approx. 10–15% of the genome is devoted to construction, dynamic architecture, sensing functions, and metabolism of the plant cell wall (Cosgrove, 2005). The major components of plant cell walls include a complex composite of polysaccharide networks, lignin (secondary walls) together with minor amounts (generally less than 10%) of *N*- and/or *O*-glycosylated proteins (Somerville et al., 2004; Cosgrove, 2005; Albenne et al., 2009; Ellis et al., 2010; Lamport et al., 2011; Zielinska et al., 2012). Protein glycosylation, through co- and/or post-translational modification, results in addition of glycans (mono-/oligo-/polysaccharides and GPI anchors) that influence a protein’s stability, location and functional properties (Lerouge et al., 1998; Nagashima et al., 2018). While *N*-glycan synthesis in the endoplasmic reticulum (ER) is relatively well conserved in eukaryotes, *N*-glycan processing and *O*-glycan biosynthesis in the Golgi apparatus (GA) are kingdom-specific and result in different oligosaccharide structures attached to glycoproteins in plants and mammals (Gomord et al., 2010). The prasinophytes situated at the base of the green plant lineage feature a much simpler set of *N*-glycan elaborations (Ulvskov et al., 2013) which may represent either the primordial eukaryotic *N*-glycosylation machinery or be the result of gene loss. Following initial processing steps in the ER, the *N*-glycans show differences in the maturation steps in the GA. Interestingly, plant N-glycans differ from their animal counterparts by the following: (1) the complete absence of sialic acid, (2) the core Fuc residues (where present) are α(1 → 3) rather than α(1 → 6)-linked to the reducing GlcNAc, and (3) the core β-mannosyl residue is often substituted with Xylβ(1 → 2) (Sturm, 1995). In contrast to *N*-glycosylation, the primary mechanism for *O*-glycosylation in plants is unique among eukaryotes and is via attachment to the hydroxyl group of the imino acid hydroxyproline (Hyp/O; in mammalian systems this type of glycosylation is to hydroxylysine) and less commonly to the hydroxyl group of serine [Ser; e.g., in extensins (EXTs) (Kieliszewski, 2001)]. This *O*-linked glycosylation determines the molecular properties and biological functions of members of the Hyp-rich glycoprotein (HRGP) superfamily and some secreted small peptides (e.g., CLE for CLAVATA3/Endosperm surrounding region). In addition, in plants there is a complete absence of GalNAc-Ser/Thr in secreted glycoproteins that is common in mammalian secreted glycoproteins and whilst there are also some other forms of *O*-glycosylation they are less common (e.g., Ser-*O*-GlcNAc on cytoplasmic and nuclear proteins). This overview should be read in conjunction with more focused reviews recently published by Seifert (2020) and Silva et al. (2020) to gain a comprehensive coverage of the structure, function and biosynthesis of arabinogalactan-proteins (AGPs).

## *N*-Glycan Processing Pathway in Plants: Glycosyltransferases (GTs) and Glycosyl Hydrolases (GHs)

**FIGURE 1:**
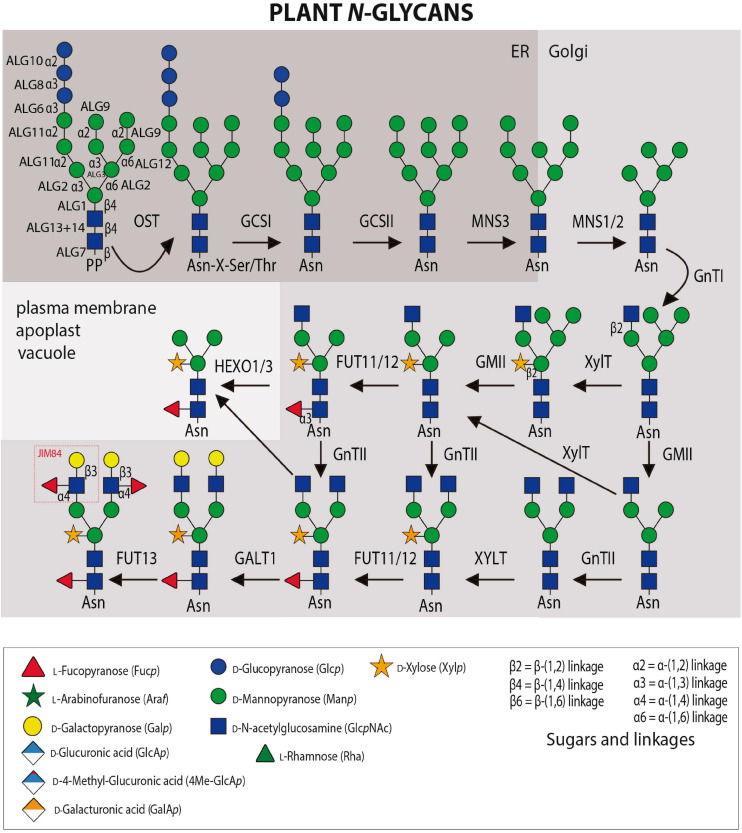
*N*-glycosylation and *N*-glycan maturation steps in plants. Several Asn-linked glycosylation (ALG) enzymes catalyze the assembly of the dolichol pyrophosphate (PP)-linked oligosaccharide in the cytosol and ER. The multi-subunit oligosaccharyltransferase (OST) complex transfers the oligosaccharide to accessible Asn-residues of nascent proteins. *N*-glycan processing enzymes: α-glucosidase I (GCSI), α-glucosidase II (GCSII), ER α-mannosidase I (MNS3), Golgi α-mannosidase I (MNS1/MNS2), β-(1 → 2)-N-acetylglucosaminyltransferase I (GnTI), β-(1 → 2)-N-acetylglucosaminyltransferase II (GnTII), β-(1 → 2)-xylosyltransferase, core α-(1 → 3)-fucosyltransferase (FUT11/FUT12), β-(1 → 3)-galactosyltransferase 1 (GALT1), and α-(1 → 4)-fucosyltransferase (FUT13). Terminal GlcNAc residues can be removed by β-hexosaminidases (HEXO1/3). GHs and GTs are listed in Tables 1, 2 while probes against *N*-glycans are listed in Table 3.

Asparagine (*N*)-linked glycosylation is a major co- and post-translational modification of proteins entering the secretory pathway. The initial step of *N*-glycosylation is the *en bloc* transfer of a preassembled oligosaccharide (Glc_3_Man_9_GlcNAc_2_) from a lipid carrier, dolicholpyrophosphate (PP-Dol) to selected Asn residues primarily in the canonical sequence Asn-X-Ser/Thr (X≠Pro) within nascent polypeptides, although some non-consensus sequences have been reported (Figure 1; Strasser, 2016). The lipid-linked oligosaccharide precursor is assembled in a stepwise manner by Asn-linked glycosylation (ALG) enzymes. The final step at the cytosolic side of the ER is catalyzed by ALG11 that transfers two consecutive α-(1 → 2) Man residues to the lipid-linked oligosaccharide. The resulting Man_5_GlcNAc_2_-PP-Dol is then transported across the ER membrane by a flippase-like protein and used as substrate in the ER lumen by the three mannosyltransferases ALG3, ALG9, ALG12 and the three glucosyltransferases (ALG6, ALG8, and ALG10) from Dol-P donors. The multi-subunit oligosaccharyltransferase (OST) complex catalyzes the transfer of the assembled oligosaccharide to the nascent polypeptide in the lumen of the ER with all subsequent steps restricted to the lumen of either the ER or GA. In the ER, the three Glc residues are sequentially trimmed by α-glucosidase I (GCSI) and II (GCSII) and a single α-(1 → 2)-Man residue is removed from the middle branch of the oligomannosidic *N*-glycan by the ER-α-mannosidase I (MNS3) to form the Man_8_GlcNAc_2_ structure (Liebminger et al., 2009). In the GA, the Golgi α-mannosidase I (MNS1/MNS2) cleaves off three additional Man residues and generates the acceptor substrate for *N*-acetylglucosaminyltransferase I (GnTI) that initiates complex-type *N*-glycan biosynthesis (von Schaewen et al., 1993; Strasser et al., 1999a, b). The product of GnTI can be either further trimmed by Golgi α-mannosidase II (GMII) or serve as a substrate for β-(1 → 2)-xylosyltransferase (XylT). N-acetylglucosaminyltransferase II (GnTII) transfers the second GlcNAc residue to complex-type *N*-glycans and core α-(1 → 3)-fucosyltransferase (FUT11/FUT12) attaches a α-(1 → 3)-linked Fuc to the innermost GlcNAc residue. The core fucosylation linkage is different from mammalian complex *N*-glycans which have a α-(1 → 6)-Fuc linked to the innermost GlcNAc residue (Strasser et al., 2004). The resulting structure (also termed GnGnXF) with two terminal GlcNAc residues, a β-(1 → 2)-linked Xyl and a core α-(1 → 3)-linked Fuc is the most prevalent complex-type *N*-glycan in plants (Wilson et al., 2001; Zeng et al., 2018). Truncated (paucimannosidic) *N*-glycans are generated in post-Golgi compartments either by the vacuolar β-*N*-acetylhexosaminidase 1 (HEXO1) or by HEXO3 which resides mainly in the plasma membrane/apoplast (Liebminger et al., 2011). The most elaborate complex-type *N*-glycans are generated in the *trans* Golgi by β-(1 → 3)-galactosyltransferase 1 (GALT1) and α-(1 → 4)-fucosyltransferase (FUT13) (Strasser et al., 2007b). The resulting Lewis A structure [α-L-Fuc*p*-(1 → 4)- β-D-Gal*p*-(1 → 3)- β-D-Glc*p*NAc-R] is ubiquitously found in plants (Fitchette-Lainé et al., 1997; Wilson et al., 2001; Zeng et al., 2018), but present only on a small number of secretory glycoproteins.

Impaired *N*-glycosylation due to either a defective OST complex or a blocked Glc removal from the transferred oligosaccharide results in lethality in *Arabidopsis* (Boisson et al., 2001; Gillmor et al., 2002; Koiwa et al., 2003; Lerouxel et al., 2005). Distinct oligomannosidic *N*-glycans in the ER are critical for ER quality control and ER-associated degradation (ERAD) (Jin et al., 2007; Hong et al., 2012; Hüttner et al., 2014). For example, the biogenesis of the *Arabidopsis* EF-Tu receptor (EFR) is dependent on Glc trimming and reglucosylation of oligomannosidic *N*-glycans and association with the lectin chaperones calnexin/calreticulin (Li et al., 2009; Lu et al., 2009). Mutant misfolded variants of BRASSINOSTEROID INSENSITIVE1 (BRI1) that expose a terminal α-(1 → 6)-linked Man on the oligomannosidic *N*-glycan are recognized by the lectin OS9 and sent to ERAD (Hong et al., 2008, 2012; Hüttner et al., 2012). Knockout of the three α-mannosidases (MNS1–MNS3) involved in trimming of oligomannosidic *N*-glycans to Man_5_GlcNAc_2_ causes a severe root development phenotype (Liebminger et al., 2009). GnTI-deficient mutants (*cgl1*) which completely lack complex *N*-glycans and display primarily Man_5_GlcNAc_2_, on the other hand, do not show any growth or morphological phenotype in *Arabidopsis* (von Schaewen et al., 1993). However, a salt sensitivity phenotype has been described for *cgl1* and other *Arabidopsis N*-glycan processing mutants that completely lack complex *N*-glycans or specific modifications in the GA and there are links to a role in cell wall formation (Kang et al., 2008). The β - (1→4)-endoglucanase KORRIGAN1, one of the potential glycoprotein candidates playing a role in these processes, does not require complex *N*-glycans for its activity (Liebminger et al., 2013), but there are other unknown factors that require GnTI and affect KORRIGAN1 function (Rips et al., 2014). Notably, an *Arabidopsis* mutant lacking complex *N*-glycans due to a deficiency in the UDP-GlcNAc transporter 1 (UGNT1) does not show a salt sensitivity phenotype (Ebert et al., 2018). Apart from *Arabidopsis*, complex *N*-glycan deficient rice and *Lotus japonicus* mutants have been characterized which display severe defects in growth and reproduction (Fanata et al., 2013; Strasser, 2014; Harmoko et al., 2016; Pedersen et al., 2017). Collectively, while the oligomannosidic N-glycans play a role in ER-quality control, the potential suite of biological functions of complex-type and paucimannosidic *N*-glycans on glycoproteins is still largely unknown and the underlying mechanisms remain to be elucidated for the described phenotypes in *Arabidopsis* and other plant species.

The biological function of the β-*N*-acetylhexosaminidase (HEXOs), especially HEXO3 acting at the plasma membrane/apoplast is unknown. In addition to HEXOs, it is possible that other GHs (Table 1) liberate monosaccharides from complex *N*-glycans either on a specific group of glycoproteins or in specific cell-types. Golgi-localized enzymes such as the recently characterized exo-β-(1 → 3)-galactosidases (Nibbering et al., 2020) may to some extent hydrolyze the Gal transferred by GALT1 directly in the Golgi (Table 2). Similarly, either *Nicotiana benthamiana* BGAL1 or another GH with β-(1 → 3/4)-galactosidase activity could modify Lewis A structures in the apoplast (Kriechbaum et al., 2020). Plant α-(1 → 3/4)-fucosidases that can cleave off Fuc residues from Lewis A structures have been identified in several plant species (Zeleny et al., 2006; Rahman et al., 2016; Kato et al., 2018). The only *Arabidopsis* GH29 α-(1 → 3/4)-fucosidase, AtFUC1, acts in the glycan degradation pathway in the vacuole and hydrolyses primarily the core α-(1 → 3)-linked Fuc. Consistent with the described substrate specificity, the AtFUC1-deficient mutant displayed slightly higher levels of Lewis A containing complex *N*-glycans. The degradation pathway for oligomannosidic and complex *N*-glycans in the vacuole involves several GHs whose substrate specificities are already well characterized (Léonard et al., 2009; Ishimizu, 2015; Kato et al., 2018). Apart from exo-glycosidases, plants have endo-glycosidases such as peptide-*N*-glycanase A (PNGase A) that is active on small glycopeptides and hydrolyzes complex *N*-glycans with core α-(1 → 3)-linked Fuc (Tretter et al., 1991; Altmann et al., 1998). Certain plant tissues such as the maize endosperm harbor an endo-glycanase (ENGase) that is active on oligomannosidic *N*-glycans and cleaves within the chitobiose core (Rademacher et al., 2008). Single GlcNAc residues or chitobiose at *N*-glycosylation sites have been detected on plant proteins (Ishimizu et al., 1999; Kim et al., 2013; Xu et al., 2016). How abundant those truncated glycans are and whether they have specific functions or represent intermediates of degradation pathways remains to be shown.

**TABLE 1:** Selected examples for carbohydrate GTs acting on *N*-glycans and *O*-glycans including type-II AGs on AGPs and EXTs from *Arabidopsis thaliana* or otherwise as indicated.

Activity	CAZy family	Protein name	References
**GTs in N-glycan processing**			
β-(1 → 2)-N-acetylglucosaminyltransferase I	13	GnTI/CGL1/GlcNAc-T1	von Schaewen et al., 1993; Strasser et al., 1999a
β-(1 → 2)-N-acetylglucosaminyltransferase II	16	GnTII	Strasser et al., 1999b
β-(1 → 2)-xylosyltransferase	61	XYLT	Strasser et al., 2000
α-(1 → 3)-fucosyltransferase	10	FUT11/FUT12	Leiter et al., 1999
β-(1 → 3)-galactosyltransferase 1	31	GALT1	Strasser et al., 2007b
α-(1 → 4)-fucosyltransferase	10	FUT13/FucTC	Wilson et al., 2001; Bakker et al., 2001
**P4Hs in AGPs/EXTs processing**			
Prolyl-4-hydroxylase	–	CrP4H1	Koski et al., 2007, 2009; Velasquez et al., 2011
		P4H2,P4H5,P4H13	Velasquez et al., 2015a
**GTs in AGPs processing**			
Hyp-*O*-galactosyltransferase	31	GALT2-GALT6; HPGT1-HPGT3 (GALT15-GALT17)	Basu et al., 2013, 2015a, b; Ogawa-Ohnishi and Matsubayashi, 2015
β-(1 → 3)-galactosyltransferase	31	GALT14 (KNS4/UPEX1)	Suzuki et al., 2017; Li et al., 2012
		GALT9 (At1g77810)	Qu et al., 2008
		GALT31A	Geshi et al., 2013; Ruprecht et al., 2020
β-(1 → 6)-galactosyltransferase	29	GALT29A	Dilokpimol et al., 2014
	31	GALT31A	Knoch et al., 2014
β-glucuronosyltransferase	14	GlcAT14A-GlcAT14E	Knoch et al., 2013; Dilokpimol et al., 2014; Lopez-Hernandez et al., 2020; Zhang et al., 2020
α-fucosyltransferase	37	FUT4	Wu et al., 2010; Liang et al., 2013; Tryfona et al., 2014
		FUT6	
		FUT7	Ruprecht et al., 2020
β-arabinosyltransferase	77	RAY1	Gille et al., 2013
**GTs in EXTs processing**			
Hyp-*O*-arabinosyltransferase	95	HPAT1-HPAT3	Ogawa-Ohnishi et al., 2013; Velasquez et al., 2015a
β-(1 → 2)-arabinosyltransferase	77	RRA1-RRA3	Egelund et al., 2007; Velasquez et al., 2011
β-(1 → 2)-arabinosyltransferase	77	XEG113	Gille et al., 2009; Velasquez et al., 2011
α-(1 → 3)-arabinosyltransferase	47	EXAD	Møller et al., 2017
Serine-*O*-galactosyltransferase	96	SGT1/SerGT1	Saito et al., 2014; Velasquez et al., 2015a

*In addition, prolyl-4-hydroxylases (P4Hs) are included. Please also see Showalter and Basu (2016) and Silva et al. (2020) for GTs acting in AGP/EXT *O-*glycan processing.*

**TABLE 2:** Selected examples for carbohydrate GHs and lyases acting on *N*-glycans, *O*-glycans including type-II AGs on AGPs and EXTs.

Activity	CAZy family	Protein name	Species	References
**GHs in N-glycan processing and degradation**				
α-(1 → 2)-glucosidase I	63	GCSI/KNF-14	*A. thaliana*	Boisson et al., 2001; Gillmor et al., 2002
α-(1 → 3)-glucosidase II	31	GCSII/RSW3	*A. thaliana*	Burn et al., 2002
ER α-(1 → 2)-mannosidase	47	MNS3	*A. thaliana*	Liebminger et al., 2009
Golgi α-(1 → 2)-mannosidase I	47	MNS1/MNS2/GMI	*A. thaliana*	Liebminger et al., 2009
			*Glycine max*	Nebenführ et al., 1999
Golgi α-(1 → 3/6)-mannosidase II	38	GMII/HGL1	*A. thaliana*	Strasser et al., 2006
α-(1 → 3/4)-fucosidase	29	FUC1	*Prunus dulcis* (almond), *A. thaliana*	Zeleny et al., 2006; Kato et al., 2018
β-(1 → 3/4)-galactosidase	35	BGAL1	*Nicotiana benthamiana*	Kriechbaum et al., 2020
β-hexosaminidases	20	HEXOs	*A. thaliana*	Strasser et al., 2007a; Liebminger et al., 2011
**Lytic enzymes acting on type-II AGs glycans of AGPs**				
FvEn3GAL	16	FvEn3GAL	*Flammulina velutipes*	Kotake et al., 2011
exo-β-(1 → 3)-galactanase	43	Il1,3Gal	*Irpex lacteus*	Tsumuraya et al., 1990; Kotake et al., 2009
	43		*A. thaliana*	Nibbering et al., 2020
endo-β-(1 → 6)-galactanase	30	Tv6GAL	*Trichoderma viride*	Kotake et al., 2004
	30	Nc6GAL	*Neurospora crassa*	Takata et al., 2010
β-(1 → 3),(1 → 6)-galactanase	35	RsBGAL1	*Raphanus sativus*	Kotake et al., 2005
β-(1 → 3),(1 → 6)-galactanase	35	SlTBG1	*Solanum lycopersicum*	Eda et al., 2014
α-L-arabinofuranosidase	54	NcAraf1	*Neurospora crassa*	Takata et al., 2010
	3	RsAraf1	*A. thaliana, Raphanus sativus* (radish), and *Bacteroides thetaiotaomicron*	Kotake et al., 2006
Bacteroides thetaiotaomicron	127			Cartmell et al., 2018
β-L-arabinopyranosidase	27	SaArap27A	*Streptomyces avermitilis*	Ichinose et al., 2009
	27	AtAPSE	*A. thaliana*	Imaizumi et al., 2017
β-glucuronidase	79	NcGlcAase	*Neurospora crassa*	Konishi et al., 2008
	79	AnGlcAase	*Aspergillus niger*	
	79	AtGUS2	*A. thaliana*	Eudes et al., 2008
4-5-anhydro-glucuronidase	154		*Bacteroides thetaiotaomicron, Bacteroides thetaiotaomicron*	Cartmell et al., 2018
	105			Cartmell et al., 2018
α-L-rhamnosidase	28	SaRha78A	*Aspergillus niger*	Martens-Uzunova et al., 2006
	78		*Streptomyces avermitilis*	Ichinose et al., 2013
	106		*Sphingomonas paucimobilis*	Miyata et al., 2005)
α-L-rhamno-glucurono lyase	PL27		*Bacteroides cellulosilyticus*	Cartmell et al., 2018
exo-α-L-(1 → 2)-fucosidase	95	AfcA	*Bifidobacterium bifidum*	Katayama et al., 2004
	95		*Aspergillus nidulans*	Pogorelko et al., 2016
	?		*Xanthomonas manihotis*	Wong-Madden and Landry, 1995
**Lytic enzymes acting on glycans in EXTs**				
β-L-arabinofuranosidase			*Bifidobacterium bifidum*	Fujita et al., 2014
β-L-(1 → 2)-arabinofuranosidase	121	HypBA2	*Bifidobacterium bifidum*	Fujita et al., 2011
β-L-(1 → 2)-arabinofuranosidase	121	XeHypBA2	*Xanthomonas euvesicatoria*	Nakamura et al., 2018
		(XCV2729)		
α-L-(1 → 3)-arabinofuranosidase	43	XeHypAA (XCV2728)	*Xanthomonas euvesicatoria*	Nakamura et al., 2018
Arabinofuranosidase-(1 → 4)-Hyp	127	XeHypBA1 (XCV2724)	*Xanthomonas euvesicatoria*	Nakamura et al., 2018

*Please also see Silva et al. (2020) for GHs acting on type-II AG *O-*glycans of AGPs.*

Oligomannosidic *N*-glycans are commonly detected with the lectin concanavalin A (ConA) derived from the jack-bean *Canavalia ensiformis* (von Schaewen et al., 1993). Complex and truncated *N*-glycans carrying β-(1 → 2)-linked Xyl and/or a core α-(1 → 3)-linked Fuc residues are detected with antibodies against horseradish peroxidase (HRP) (Wilson et al., 1998; Strasser et al., 2004). The Lewis A structure is specifically recognized by the monoclonal antibody (mAb) JIM84. Bacterial endo-β-N-acetylglucosaminidase H (Endo H) cleaves within the unsubstituted chitobiose core to release oligomannosidic N-glycans from glycoproteins (Tarentino et al., 1974). In contrast to the PNGase A from almond, PNGase F from *Flavobacterium meningosepticum* is inhibited by the presence of core α-(1 → 3)-linked Fuc (Tretter et al., 1991) and therefore only of limited use for the deglycosylation of plant glycoproteins decorated with complex *N*-glycans.

## *O*-Glycans, GTs and GHs of Plant Glycoproteins

*O*-linked glycosylation defines the molecular properties and biological function of the HRGP superfamily and some secreted small hormone peptides (e.g., CLE-like peptides). The HRGP superfamily is traditionally divided into three major subgroups: AGPs, EXTs including the Leucine-Rich eXtensins (LRXs), and the repetitive Pro-rich proteins (PRPs) (Seifert and Roberts, 2007; Ellis et al., 2010; Tan et al., 2012; Hijazi et al., 2014; Johnson et al., 2017). However, the HRGP superfamily is better understood as a spectrum of molecules ranging from the highly glycosylated AGPs to the minimally *O*-glycosylated PRPs. Two major types of *O*-glycans are attached to Hyp (O) in plant glycoproteins. The first type includes unbranched chains of up to five arabinose (Ara) units added to clusters of Hyp residues in EXTs (Marzol et al., 2018) and small CLE-like peptides (Ohyama et al., 2009; Shinohara and Matsubayashi, 2013). The second type are complex type II arabino-3,6-galactans (AGs) which are attached to non-contiguous Hyp residues (AO/SO/TO/VO) on AGPs and AGP-like proteins (Johnson et al., 2017). Finally, a single Gal is linked to Ser mostly in EXTs and EXT-related proteins. The Hyp contiguity hypothesis proposes that the addition of these two main types of *O*-glycan is controlled by “glycomotifs” in the HRGP protein sequence (Kieliszewski, 2001). This hypothesis predicts that short arabino-oligosaccharides are added to contiguous Hyp_3__–__5_ residues in EXTs, whereas complex AGs are assembled on clustered but non-contiguous Hyp residues in AGPs (Shpak et al., 1999; Tan et al., 2010). The only exception to this rule is CLE-like peptides (e.g., Tob/Tom-HypSys, PSY1, CLV3, and CLE2), in which non-contiguous Hyp residues are arabinosylated (Ohyama et al., 2009; Shinohara and Matsubayashi, 2013). The extent of glycosylation of PRPs remains unclear with low levels of Ara residues presumably *O-*linked to Hyp (Bernhardt and Tierney, 2000).

### Arabinogalactan-Proteins-*O*-glycans and GTs

**FIGURE 2:**
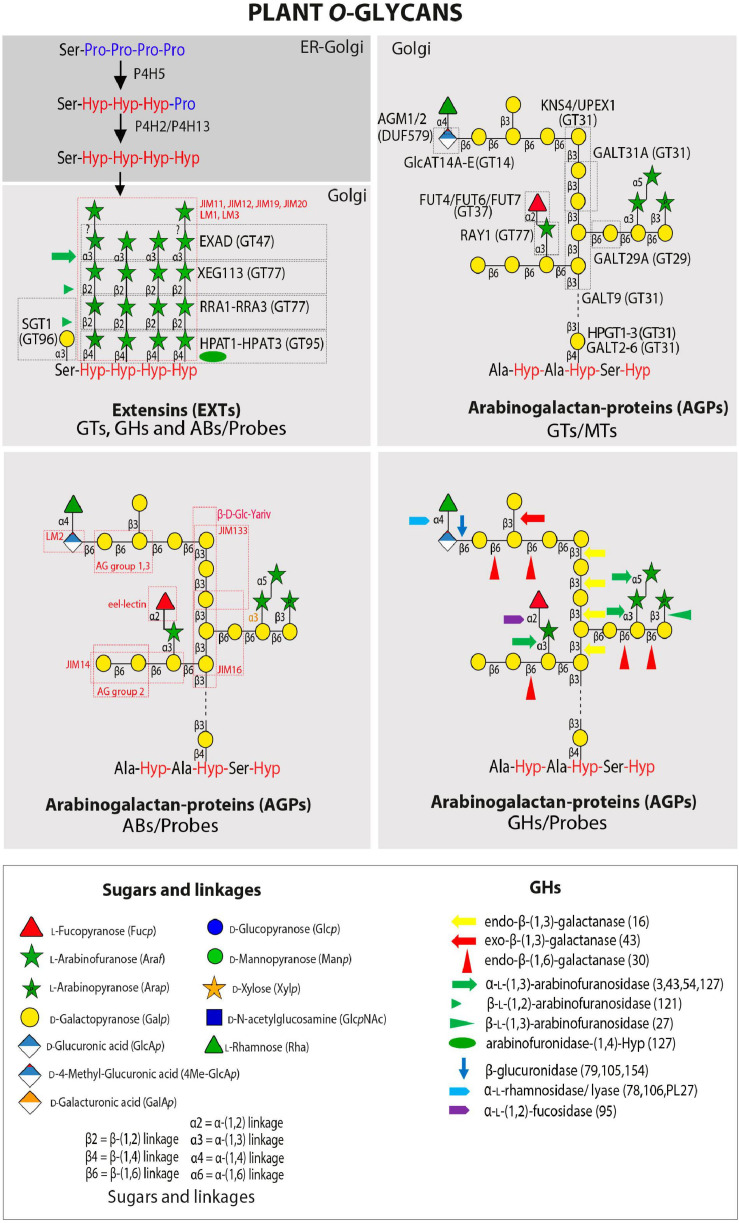
Plant *O*-glycans. Schematic representation of an average carbohydrate structure of EXTs and AGPs with the GTs and GHs that have been characterized to date. Illustrated are the complex sugar side chains and the different linkages that are found in the sugar backbone. GHs and GTs are listed in Tables 1, 2 while probes recognizing specific epitopes in AGPs and EXTs are listed in Table 3. Number in brackets refer to GHs CAZY family. Please also see Silva et al. (2020) for GTs and GHs acting in AGP *O-*glycan processing.

Arabinogalactan-proteins are complex cell surface proteoglycans with type II AG glycan moieties attached at non-contiguous Hyp residues consisting of a β-(1 → 3)-galactan backbone substituted at C(*O*)6 with side chains of β-(1 → 6)-galactan of variable length decorated further with Ara, and less frequently also with Fuc, Rha, (*O*-methyl)glucuronic acid (4-*O*-MeGlcA) and Xyl (Figure 2). AGPs have been implicated in a diverse array of plant growth and development processes including hormone signaling, cell expansion and division, embryogenesis of somatic cells, differentiation of xylem, reproduction and responses to abiotic stress (Seifert and Roberts, 2007; Ellis et al., 2010; Ma et al., 2018). Recently, it was shown that perturbing an AG-peptide (AGP21) in *Arabidopsis* triggers aberrant root hair development by altering expression of the homeodomain protein GLABRA 2 (GL2) expression in a BIN2 (a Type-II GSK3-like kinase)-dependent manner, similar to the phenotype observed in plants with defective brassinosteroid signaling (Borassi et al., 2020). These results imply an interesting parallel between plant AGPs and animal heparin sulfate proteoglycans (HSPGs), which are important co-receptors in signaling pathways mediated by growth factors, including members of Wnt/Wingless, Hedgehog, transforming growth factor−β, and fibroblast growth factor family members (Lin, 2004). AGP4, AGP6, and AGP11 from *Arabidopsis* have been shown to be essential for reproduction, with AtAGP4 shown to play a critical role in synergid degeneration and prevention of more than one pollen tube being attracted to the embryo sac (Pereira et al., 2016). AG glycan structures have also been found to be involved in reproductive development in *Torenia fournieri* with a methyl-glucuronosyl arabinogalactan (AMOR) released from the ovule inducing the competency of the pollen tube to respond to ovular attractant peptides (Mizukami et al., 2016; Jiao et al., 2017). UPEX1/KNS4/GALT14, a galactosyltransferase (GALT) from *Arabidopsis* that generates the β-(1 → 3)-galactan backbone of type II AG, has been shown to be vital for normal pollen exine development as *upex1/kns4/galt14* mutants display a collapsed pollen phenotype with reduced viability and fertility (Suzuki et al., 2017). The requirement for specific glycan structures on AGPs for Ca^2+^ signaling during development is supported by mutants in GlcAT14 members. AG glycans with reduced glucuronosylation were shown to have lower Ca^2+^ binding capacity (Lopez-Hernandez et al., 2020). Double/triple *glcat* mutants displayed developmental defects that could be suppressed by additional Ca^+2^ in growth media. Unique glycan structures on AGPs in seagrasses, that include a high content of terminating 4-*O*-methyl-GlcA residues, are proposed to strengthen Ca^2+^ binding and limit the effects of salt as an adaptation to the marine environment (Pfeifer et al., 2020). These few examples demonstrate the indispensable nature of AGPs to plant processes and the important function their *O*-glycan moieties play, although their mechanistic role continues to remain elusive and ill-defined as recently reviewed (Seifert, 2020).

In *Arabidopsis*, *O*-glycosylation of AGPs is initiated by a set of 8 Hyp-galactosyltransferases (Hyp-GALTs/HPGTs), which are members of the GT31 family^1^ (Lombard et al., 2014) and designated as GALT2-GALT6 and HPGT1-HPGT3 (also designated as GALT15-GALT17, respectively) (Basu et al., 2015a, b; Ogawa-Ohnishi and Matsubayashi, 2015) by different groups (Table 1 and Figure 2). These enzymes add a single Gal unit to Hyp residues. Other known GTs include β-(1 → 3)-GalTs also from the GT31 family such as GALT8, GALT9, KNS4/UPEX1/GALT14, (Qu et al., 2008; Li et al., 2012; Suzuki et al., 2017; Ruprecht et al., 2020) and β-(1 → 6)-galactosyltransferases such as GALT29A from GT29 (Geshi et al., 2013; Dilokpimol et al., 2014) although this activity is yet to be independently verified. Previously reported β-(1 → 6)-GalT activity for GALT31A (Geshi et al., 2013) has not been confirmed, rather it has been shown to possess β-(1 → 3)-GalT activity (Ruprecht et al., 2020). β-Glucuronosyltransferases including GlcAT14A-GlcAT14E from the GT14 family (Knoch et al., 2013; Dilokpimol et al., 2014; Lopez-Hernandez et al., 2020; Zhang et al., 2020) have been characterized as well as α-fucosyltransferases (FUT4, FUT6, and FUT7 from GT37) (Liang et al., 2013; Tryfona et al., 2014, Ruprecht et al., 2020) and a β-arabinosyltransferase (Reduced Arabinose Yariv1/RAY1 from GT77) (Gille et al., 2013). Two GlcA methyltransferases (AtAGM1 and AtAGM2) have also recently been identified (Temple et al., 2019). Collectively, this body of work highlights that robust data are required to confidently assign biochemical function(s) to GTs. Several other GTs involved in type II AG biosynthesis remain to be identified, including β-(1 → 6)-GalTs that elongate the side chains and other arabinosyltransferases, rhamnosyltransferases, and xylosyltransferases that decorate the non-reducing termini of the galactan chains as well as additional sugar modifying enzymes. Furthermore, structural characterization using NMR of artificial AGPs expressed in tobacco cell suspension cultures indicated kinks of β-(1 → 6)-linked Gal in the β-(1 → 3)-galactan backbone, suggesting the existence of additional GTs catalyzing the synthesis of this linkage (Tan et al., 2004, 2010). Please also see a recent review by Silva et al. (2020) that has reviewed the GTs acting in AGP *O-*glycan processing.

### Variability of Type II AG *O*-Glycans

**FIGURE 3:**
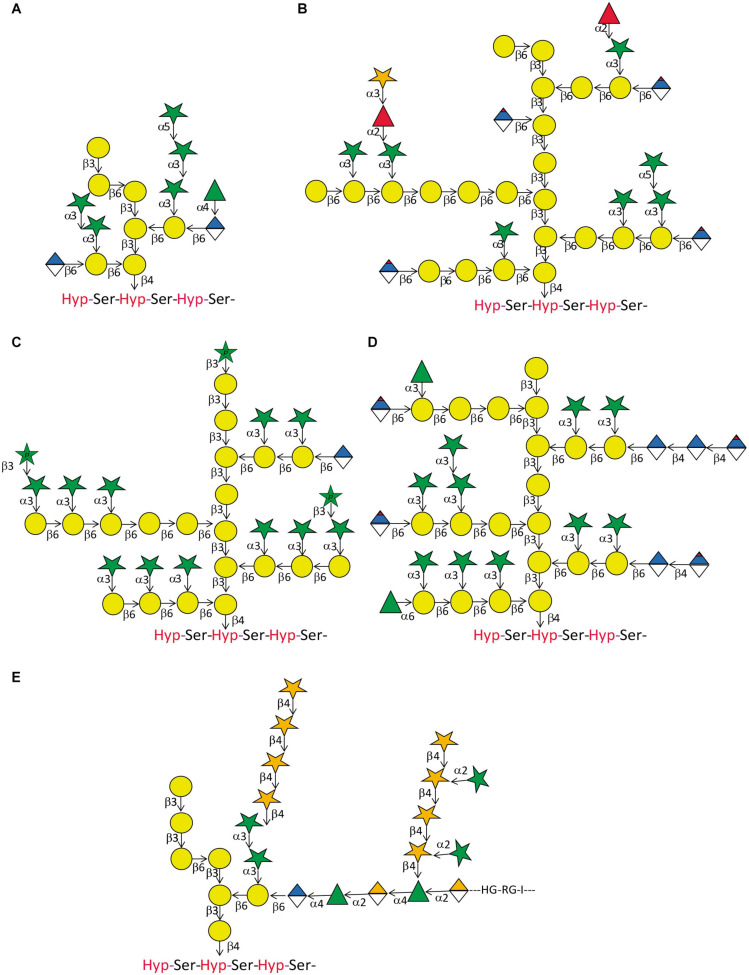
Arabinogalactan-protein glycan variation. Five structures of type II glycans found on AGPs demonstrating common motifs and variations. **(A)** This relatively small glycan was produced on an artificial AGP recombinantly expressed in tobacco cell cultures by Tan et al. (2004). Note the β-(1 → 6) kink in the β-(1 → 3) galactan backbone. **(B)** This structure approximates the model described for AGP glycans purified from *A. thaliana* leaves (Tryfona et al., 2012). Note that the actual size of many of the glycans is probably much bigger than the structure displayed here. In a later study by the same group, the terminal modification of L-Fuc by D-Xyl was described (Tryfona et al., 2014). **(C)** Using the same tools of enzymatic degradation and mass spectrometry, this group also described the glycan-structure of wheat flour AGP (Tryfona et al., 2010). Again, we show an approximation of their model that should accommodate large variations in glycan size. A noteworthy feature of this glycan is the occurrence of terminally linked L-Ara*p*. **(D)** AGP-glycans of the see grass *Zostera marina* are particularly rich in 4-Me-GlcA*p* (Pfeifer et al., 2020). **(E)** The partial glycan structure of the type II AG linked to an AGP named as APAP1 that is linked to both rhamnogalacturonan 1 (RG1) and arabinoxylan (AX) (Tan et al., 2013). Legend for sugar symbols is as per Figure 2.

Compared to the relatively high degree of conservation of *N*-glycan structures, *O*-glycans attached to AGPs display a considerable degree of variation on every level (Figure 3 and references there in). There are variations between different species and tissues and in the same cell type at different stages of development. The common structural feature of type II AG that are *O-*linked to isolated Hyp residues on AGPs is a backbone of β-(1 → 3) Gal that contains β-(1 → 6) linked Gal side chains of variable length, although there are examples of β-(1 → 6) linked Gal backbones (Raju and Davidson, 1994; Dong and Fang, 2001). In some reports a β-(1 → 6) linked Gal is further β-(1 → 3) galactosylated forming a kink in the backbone (Churms et al., 1983; Bacic et al., 1987). Mostly however, the Gal side chains are modified by α-(1 → 3) linked L-Ara*f* (Tryfona et al., 2010, 2012 and references therein). Additionally, the side branches can contain β-(1 → 6) linked GlcA or 4-*O*-MeGlcA. The L-Ara*f* side groups are sometimes extended by one or two α-(1 → 3) linked L-Ara*f* residues and terminated by either α-(1 → 3) linked L-Ara*f* or α-(1 → 2) linked L-Fuc. In some cases, the L-Fuc is not the terminal sugar but further modified by β-(1 → 3) linked D-Xyl. While L-Ara*f* incorporated in plant cell wall carbohydrates is predominantly found in its furanose form there have also been reports on L-Ara*p* β-(1 → 3) linked to Ara*f* or Gal*p* as terminal sugars. Likewise, GlcA*p* and 4-*O*-methyl D-GlcA*p* are often found as terminal modifications of the galactan backbone but sometimes GlcA was found decorated by α-(1 → 4) linked L-Rha. In other cases, another β-(1 → 4) linked D-GlcA followed and terminated by β-(1 → 4) linked 4-*O*-methyl D-GlcA*p* were linked to this sugar. Another modification of D-GlcA*p* was α-(1 → 4) linked L-Rha as the first sugar of an extended heteropolymer resembling rhamnogalacturonan I. Besides this staggering multitude of structures attributed to AGP-linked type II AG, there exists variability in the degree of substitution of individual Hyp residues as well as the sizes of the individual glycans. This was demonstrated for artificial AGP-like fluorescent proteins that showed considerable variations in apparent molecular weight between different organs (Estevez et al., 2006). Moreover, the cell-type specific variation between type II AG structures is elegantly revealed by AGP-glycan specific monoclonal antibodies (mAbs) (Table 3).

**TABLE 3:** Toolkit Abs/probes available to characterize *N*- and *O*-glycans.

mAbs/reagent	Species origin	Tissue origin	Minimal epitope recognized	References
JIM4	*Daucus carota*	Suspension cultured cells	β-GlcA-(1 → 3)-α-GalA-(1 → 2)-Rha	Knox et al., 1989; Yates et al., 1996
JIM8	*Beta vulgaris*	Suspension cultured cells	unknown	Pennell et al., 1991
JIM13	*Daucus carota*	Suspension cultured cells	β-GlcA-(1 → 3)-α-GalA-(1 → 2)-Rha	Knox et al., 1991; Yates et al., 1996
JIM14	*Daucus carota*	Suspension cultured cells	β-Gal-(1 → 6)-β-Gal-(1 → 6)-β-Gal-(1 → 6)	Knox et al., 1991; Yates et al., 1996; Ruprecht et al., 2017
JIM15	*Daucus carota*	Suspension cultured cells	unknown	Knox et al., 1991; Yates et al., 1996
JIM16	*Daucus carota*	Suspension cultured cells	β-Gal-(1 → 6)	Knox et al., 1991; Yates et al., 1996; Ruprecht et al., 2017
			β-Gal-(1 → 3)-β-Gal-(1 → 3)-β-Gal-(1 → 3)	
JIM84	*Daucus carota*	Suspension cultured cells	α-L-Fuc*p*-(1 → 4)-β-D-Gal*p*-(1 → 3)-β-D-Glc*p*NAc-R	Horsley et al., 1993
JIM101	*Gymnocolea inflata*	Extracted AGPs	unknown	Pattathil et al., 2010
JIM133	*Zinnia elegans*	Tracheary element cell walls	β-Gal-(1 → 3)-β-Gal-(1 → 3)-β-Gal-(1 → 3)	Pattathil et al., 2010; Ruprecht et al., 2017
LM2	*Oryza sativa*	Suspension cultured cells	β-GlcA-(1 → 6)-β-Gal-(1 → 6)-β-Gal-(1 → 6)-β-Gal-(1 → 6)	Smallwood et al., 1996; Ruprecht et al., 2017
LM14	*Arabidopsis thaliana*	Mixed leaves, stems and roots	Unknown	Møller et al., 2008
MAC204	*Pisum sativum*	Peribacteroid membrane	Unknown	Bradley et al., 1988
MAC207	*Pisum sativum*	Peribacteroid membrane	β-GlcA-(1 → 3)-α-GalA-(1 → 2)-Rha	Pennell et al., 1989; Yates et al., 1996
PN16.4B4	*Nicotiana glutinosa*	Suspension cultured cells	Unknown	Norman et al., 1986; Pattathil et al., 2010
β-Glc Yariv (synthetic dye)			β-Gal-(1 → 3)-β-Gal-(1 → 3)-β-Gal-(1 → 3)-β-Gal-(1 → 3)-β-Gal)_*n*__>__5_	Yariv et al., 1967; Kitazawa et al., 2013; Paulsen et al., 2014

### Arabinogalactan-Protein-Glycans Probes, Abs, and GHs

Characterization of AGP glycan structures is difficult due to the enormous diversity of protein backbones, the difficulty in extracting and purifying individual AGPs and the heterogeneity in their glycan moieties (Tan et al., 2012; Johnson et al., 2017). NMR techniques require significant amounts of relatively homogeneous samples and are therefore only rarely used on natural AGP glycans; for example, on the AGP glycans from pistils of *Nicotiana alata* (Gane et al., 1995). The most successful approaches include performing (i) linkage analyses of glycans in combination with partial chemical degradation of the polysaccharides (Pfeifer et al., 2020) and (ii) partial enzymatic degradation (GHs; Table 2) to analyze resulting oligosaccharides using carbohydrate gel electrophoresis (PACE) and MS fragmentation techniques (Tryfona et al., 2010, 2012). For specific detection of AGPs, β-Glc Yariv phenylglucoside reagent (1,3,5-tri-(4-β-D-glucopyranosyl-oxyphenylazo)-2,4,6-trihydroxybenzene) that recognizes and binds to β-(1 → 3)-Gal-linked oligosaccharides, six residues or longer, is used. In addition, numerous mAbs against AGP glycan epitopes are available (Table 3). A group of 36 AGP mAbs recognize a core set of Gal-β-(1 → 6)-Gal epitopes that are further sub-divided into three groups defined by side branches permitted and forbidden for mAb binding (Figure 2; Pattathil et al., 2010; Ruprecht et al., 2017). In addition, some mAbs recognize different epitopes, namely a β-(1 → 3)-trigalactosyl glycan (JIM133), a β-(1 → 6)-trigalactosyl glycan (JIM14) or a β-(1 → 6)-Gal branched β-(1 → 3)-trigalactosyl glycan (JIM16) while the terminal β-(1 → 6)-glucuronosyl modification is recognized by LM2. Furthermore, eel lectin binds to the terminal α-(1 → 2)-L-Fuc residue modification on AGPs. The binding epitopes of several AGP-specific mAbs remain to be characterized (e.g., JIM4, JIM8, JIM13, MAC207, and LM14) (Table 3).

An as yet incomplete list of GHs acting on various linkages in type II AGs are mainly known from various microbial sources (Table 3) and are used for their structural characterization. However, plant endogenous AGP-specific GHs have also been described. Two family GH43 exo-β-(1 → 3)-galactanases from *Arabidopsis* were shown to be required for controlling the apparent abundance of AGPs and their loss of function resulted in a sugar-conditional root expansion phenotype characteristic of many primary cell wall-defective mutants (Nibbering et al., 2020). *Arabidopsis* also has three close homologs encoding family 79 GHs. One member of this family named AtGUS2 was identified in a gel filtration fraction that showed *O*-β-glucuronidase activity *in vitro* (Eudes et al., 2008). A T-DNA insertion in this locus displayed abnormally short hypocotyls and overexpression of AtGUS2 enhanced both hypocotyl length and root length with purified AGPs displaying lower terminal-GlcA content. Finally, four *Arabidopsis* loci encode family 27 GHs named β-L-ARAPASE (APSE), and α-GALACTOSIDASE 1-3 (AGAL1-3) (Imaizumi et al., 2017). Although the majority of L-Ara found in plant carbohydrates is in its furanose form some examples of L-arabinopyranose (Ara*p*) exist, one example being found in type II AGs (Tryfona et al., 2010). It was suggested that APSE and the AGALs act on these residues (Imaizumi et al., 2017), and *apse agal3* mutants showed decreased β-L-arabinopyranosidase activity and increased levels of β-L-Ara*p*, compared to wild type. Apart from a decrease in hypocotyl length, the *apse agal3* mutants appeared phenotypically normal. Finally, a promiscuous α-L-arabinofuranosidase/β-D-xylosidase belonging to family GH3 has been purified and cloned from radish (Kotake et al., 2006]. However, since the Ara and Xyl residues exist in various carbohydrates it is presently unknown whether any of the fifteen *Arabidopsis* GH3 enzymes act as AGP-specific α-L-arabinofuranosidases. In addition, please see Silva et al. (2020) for GHs, both endogenous and heterologous, acting on type-II AG glycans of AGPs.

### Extensins-Glycans, GTs, and GHs

Extensins are characterized by repetitive Ser-Hyp_3__–__5_ repeats, where the contiguous Hyp residues are substituted with up to 4–5 units of L-Ara*f* with the following structure Hyp-*O*-(4 → 1)-β-L-Ara*f*-(2 → 1)-β-L-Ara*f*-(2 → 1)-β-L-Ara*f*-(3 → 1)-α-L-*term* Ara*f*; the linkage of the fifth Ara residue is not yet resolved (Velasquez et al., 2011; Møller et al., 2017), and the Ser is substituted with D-Gal as Ser-*O*-(1 → 3)-α-Gal*p* (Saito et al., 2014) (Figure 2). EXTs and secreted signaling peptides require the conversion of specific peptidyl-proline residues to *trans*-4-Hyp by prolyl-4-hydroxylase (P4H) enzymes (Table 1). P4H enzymes are 2-oxoglutarate (2OG) dioxygenases that catalyze the formation of *trans*-4-Hyp from peptidyl-Pro (Koski et al., 2007, 2009). In root cells, P4H5 is the main P4H that initiates the hydroxylation of some Pro residues in EXTs, whereas P4H2 and P4H13 complete the hydroxylation on these contiguous Pro residues (Velasquez et al., 2011, 2015b). The first Ara*f* is added by Hyp-*O*-β-arabinosyltransferase 1-3 (HPAT1-HPAT3), which belong to the GT95 family (Ogawa-Ohnishi et al., 2013). *Reduced Residual Arabinose 1–3* (RRA1-RRA3) enzymes of the GT77 family are thought to transfer the second Ara*f* (Egelund et al., 2007; Velasquez et al., 2011), while the third residue addition is catalyzed by Xyloglucanase113 (XEG113), which also belongs to the GT77 family (Gille et al., 2009). XEG113 was first identified in a screen of mutagenized *Arabidopsis* plants subjected to growth in liquid media in the presence of a xyloglucanase with *xeg113* plants exhibiting more elongated hypocotyls than WT, providing genetic evidence that extensin arabinosylation is important for cell elongation (Gille et al., 2009). Finally, *Extensin Arabinose Deficient* (ExAD) transfers the fourth Ara*f* residue with a α-(1 → 3) linkage. ExAD belongs to clade-E of the inverting GT47 family (Møller et al., 2017) (Table 1). The arabinosyltransferase that adds the fifth and final Ara unit has not yet been identified (Velasquez et al., 2011). On the other hand, *O*-arabinosylation with β-linked-L-arabinofuranosyltransferases at Hyp also takes place in the short signaling peptides of the CLE-like family using identical linkages/stereochemistry as used for the innermost three Ara*f* residues found in the EXTs (Ito et al., 2006; Ohyama et al., 2009; Matsuzaki et al., 2010), suggesting that similar P4Hs and GTs might participate in these post-translational modifications. A single Serine-galactosyltransferase (SGT1/SerGT1) adds Gal to Ser in the repeated Ser-Hyp_3__–__5_ motif in EXTs (Saito et al., 2014). SerGT1 is the first example of a GT in the context of protein glycosylation with type-I membrane protein topology (i.e., N-terminal catalytic domain within the Golgi lumen) with no homology to known GTs, indicating that it is a novel plant-specific GT of the GT96 family (Table 1). Several EXT-specific mAbs are used to detect EXT epitopes but these epitopes remain to be structurally characterized (e.g., JIM11, JIM12, JIM19, JIM20, LM1) (see Table 3; Rydahl et al., 2018 and references therein).

Several GHs from different bacterial sources have been described that hydrolyze specific linkages within *O*-glycans of EXTs (Table 2). The GH127 enzyme from *Xanthomonas euvesicatoria* XeHypBA1 was described as a β-L-(1 → 2)-arabinofuranosidase (Nakamura et al., 2018) while two GH121 members, one from *Bifidobacterium bifidum* HypBA2 and XeHypBA2, were shown to hydrolyze the β-L-Ara*f*-(2 → 1) linkages (Fujita et al., 2011, 2014; Nakamura et al., 2018). Finally, an α-L-(1 → 3)-arabinofuranosidase XeHypAA is able to hydrolyze β-L-Ara*f*-(3 → 1)-α-L-Ara*f* (Nakamura et al., 2018) (Table 2 and Figure 2). It is unclear if endogenous β-arabinofuranosidases are encoded by plant genomes, and if so whether they are secreted into the apoplast to regulate the length of EXTs *O*-glycans.

### Decoding EXTs and Their *O*-Glycans Functions

It is already known that *O*-glycans increase HRGP solubility, resistance to proteolytic degradation and thermal stability (Shpak et al., 2001; Kieliszewski et al., 2011; Lamport et al., 2011; Seifert, 2020). EXTs are able to form, at least *in vitro*, a tridimensional covalent network through diTyr-linkages mediated by EXT peroxidases between individual EXT molecules and also via self-recognition and alignment of hydrophilic O-glycosylated Ser-(Hyp)_3__–__4_ repeats and hydrophobic peptide-cross-linking modules (Cannon et al., 2008). Thus, the ordered EXT monomer assembly in plant cell walls would involve a zipper-like endwise association via cross-linking at the ends of the molecules (Kieliszewski et al., 2011; Lamport et al., 2011). Recently, modeling experiments suggested that classical EXTs would be able to form a putative triple helix structure by lateral staggered alignment (Cannon et al., 2008) and diTyr cross-linking, similar to that present in collagen (Velasquez et al., 2015b; Marzol et al., 2018). It is also proposed that EXTs interact with pectins by a simple acid-base reaction forming a supramolecular ionic structure in the nascent cell wall (Valentin et al., 2010), which would serve as a framework for further cell wall deposition (Cannon et al., 2008; Lamport et al., 2011). In addition, covalent EXT-pectin cross-links were also suggested (Nuñez et al., 2009). However, it is unclear how EXT monomers are secreted and assembled into the glyco-network and how EXT and related glycoproteins-pectin interactions are controlled in a coordinated way during new cell wall formation.

Several mutants in *O*-glycosylation GTs of EXTs and related proteins (e.g., LRXs) have similarities to root hair-defective growth phenotypes (Velasquez et al., 2011; 2015b) and EXT content and their *O*-glycosylation levels were correlated with cotton fiber cell elongation (Guo et al., 2019), highlighting that *O*-glycans in EXTs affect EXT function during plant cell expansion. Furthermore, an *in vitro* study has revealed that both Ser-O-galactosylation and Hyp-O-arabinosylation determine the rate of EXT crosslinking and hence the efficiency of EXT network formation (Chen et al., 2015). Thus, correct arabinosylation of EXTs is essential for their *in vivo* functions. In addition, some of these mutants (e.g., *rra2* and *xeg113*) showed enhanced susceptibility for specific root pathogens (Castilleux et al., 2020). The known roles of EXTs in cell wall assembly, cell shape and growth raises the question to the function of each individual EXT molecule (Hall and Cannon, 2002; Cannon et al., 2008; Velasquez et al., 2011). Although the *Arabidopsis* genome encodes several EXTs, so far only a single EXT mutant *rsh* (for *root shoot hypocotyl-defective*)*/ext3*) have a nearly lethal phenotype (Cannon et al., 2008). This finding suggests either the high redundancy or masked functions of EXTs in plant development, although their role in root hairs, pollen tubes and root growth are clear exceptions to this rule. Several EXT mutants (*ext6-7/12-14/18*) (Velasquez et al., 2011) and *lrx1/2* mutants have aberrant root hair morphologies (Baumberger et al., 2001, 2003a, b; Ringli, 2010) and *prp3* (Bernhardt and Tierney, 2000) display short root hairs. Characterization of multiple mutants for pollen LRXs (*lrx8/9/10/11*) indicates they are key components for proper polar growth as sentinels of cell wall integrity in these rapidly expanding cells (Fabrice et al., 2018; Wang et al., 2017; Sede et al., 2018; Herger et al., 2019) while the triple mutant *lrx3/4/5* showed defects in cell expansion in root cells (Draeger et al., 2015), possibly mediated by abnormal vacuolar expansion (Dünser et al., 2019). Recently, a mechanism of action for LRXs was proposed based on LRX8 and LRX9 binding in the apoplast to the Rapid Alkalinization Factor 4-19 (RALF4 and RALF19) peptides as well as to the extracellular domains of some transmembrane receptors such as CrRLK1Ls (e.g., ANX1,2 and BUDS1,2) (Ge et al., 2017; Mecchia et al., 2017). In a similar manner, the extracellular LRX3/4/5-RALF22/23 together with CrRLK1L FERONIA (FER) are able to coordinate growth under salt conditions (Zhao et al., 2018, 2020) and LRX1/5-RALF1-FER in shoot and root growth (Dünser et al., 2019; Herger et al., 2020). It has been proposed that LRXs work together with CrRLK1Ls and RALF peptides to monitor the plant cell wall integrity status during cell growth (Ge et al., 2017; Mecchia et al., 2017; Dünser et al., 2019; Herger et al., 2020). Although the structural basis for the interaction between LRXs and RALFs peptides was recently established (Moussu et al., 2020), it is unclear how the *O*-glycans in the EXT domain of LRXs affects these protein-protein interactions. Since the EXT domain is variable among LRXs both in terms of length and motif (Baumberger et al., 2003a, b; Borassi et al., 2016), it is proposed that it has adapted to the specific cell wall architecture of the numerous tissues where they are located as putative cell wall integrity sensors (Baumberger et al., 2003a, b; Marzol et al., 2018; Sede et al., 2018; Herger et al., 2019).

## Chemical Synthesis, Glycan Arrays and Technological Challenges

**FIGURE 4:**
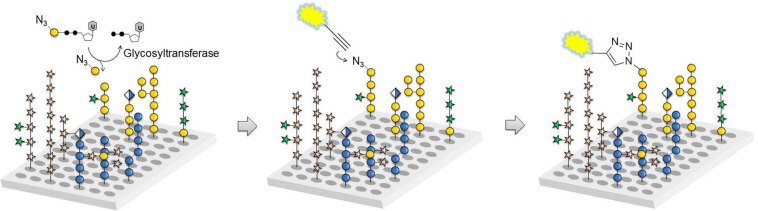
Glycan array assay for GT characterization. Glass slides equipped with plant cell wall-related oligosaccharides are incubated with azido-functionalized sugar nucleotides and GT candidates expressed in, for example human embryonic kidney (HEK) 293 cells. Any transferred monosaccharide is visualized by azide-alkyne cycloaddition reaction with a fluorescent dye to determine reactive acceptors (reprinted from Ruprecht et al., 2020).

The tremendous heterogeneity of plant cell wall glycans such as the *O*-glycans in AGPs make the identification of the exact molecular structures that serve either as acceptors for GTs, substrates for GHs or epitopes for mAbs very challenging. There are basically two options to procure suitable oligosaccharide samples for biochemical assays used in GT functional studies. One possibility is purification of oligosaccharides from digests of natural polysaccharides or glycoproteins, which can provide a large number of oligosaccharides in acceptable time, but oftentimes with compromised purity and in limited quantities (Tan et al., 2012). The second possibility is chemical synthesis, which gives access to significant amounts of well-defined and pure oligosaccharides but is very time consuming (Kinnaert et al., 2017; Pfrengle, 2017). Automated glycan assembly (AGA) can significantly accelerate the process of chemical synthesis for a number a glycan classes (Seeberger, 2015). In AGA, protected monosaccharide building blocks are coupled in a stepwise manner to a linker-functionalized Merrifield resin, in a computer-controlled and automated manner. While many different complex oligosaccharides have been synthesized by AGA, only recently has it begun to be explored for synthesizing plant glycans, including AGP *O*-glycans (Bartetzko and Pfrengle, 2019). Chemically synthesized glycans as well as natural polysaccharides and isolated oligosaccharides can be printed as glycan arrays to obtain high-throughput platforms for analyzing plant cell wall-related enzymes and molecular probes such as mAbs (Møller et al., 2008; Pedersen et al., 2012). A recently developed glycan array equipped with chemically synthesized plant cell wall glycans, including many AGP glycan related substrates, has proven useful for the rapid characterization of a large number of cell wall glycan-directed mAbs (Ruprecht et al., 2017). The same glycan array has also aided in identifying acceptor substrates for GTs involved in AGP glycan biosynthesis such as GalT31A and FUT7 (Figure 4; Ruprecht et al., 2020). By extension, this technology has the potential to reveal the biochemical function of novel GTs that act in the *O*-glycosylation pathway of plant HRGPs and other glycoproteins.

## Perspectives and Future Challenges in Plant Glycobiology

Major developments in nuclease-based gene editing, quantitative transcriptomics, metabolomics, and proteomics are now enabling high throughput approaches to explore plant protein and lipid glycosylation through analyzing and targeting enzymes involved in glycosylation processes. Although there has been significant progress in plant glycobiology, there are still many remaining fundamental questions to be addressed. Here, we attempt to highlight some selected aspects that are key to accelerating progress in this field:

• *In vivo N- and O-glycan mapping*. The chemical reporter strategy known as bio-orthogonal click chemistry has arisen as a powerful methodology to investigate the dynamics and functions of non-genetically encoded biomolecules such as sialylated (Chang et al., 2009; Laughlin and Bertozzi, 2009; Mbua et al., 2013), fucosylated (Hsu et al., 2007; Laughlin and Bertozzi, 2009; Besanceney-Webler et al., 2011), and mucin-type *O*-linked glycans (Laughlin and Bertozzi, 2009; Baskin et al., 2010) in live cells and model organisms (Prescher and Bertozzi, 2005; Grammel and Hang, 2013). This approach relies on the labeling of specific sugars by feeding cells with a synthetic monosaccharide analog carrying a chemical reporter that is then reacted with a probe (e.g., a fluorophore suitable for fluorescent microscopy imaging) in living systems to locate/visualize the incorporated reporter. Despite the fast-growing number of examples of this potent method in animal cells, reports describing its use in plant biology are surprisingly few (Anderson et al., 2012; Dumont et al., 2016; Zhu et al., 2016; Zhu and Chen, 2017). In part, this is due to the capacity of these probes to penetrate the cell wall barrier and, in part, due to the limited diversity of sugar analogs available to replace the endogenous sugars that need to be transported into the plant cell, and incorporated into glycan structures by GTs in a similar manner. Other new technologies are being developed to directly perform imaging of single glycan molecules that are isolated by mass-selective, soft-landing electrospray ion beam deposition and imaged by low-temperature scanning tunneling microscopy (Wu et al., 2020). This generates glycan structures at the single-molecule and single cell levels to directly relate how molecular structure correlates with properties – a step forward toward cracking the “sugar code.”
• *GT activity characterization by glycan arrays.* The use of glycan arrays equipped with oligosaccharide acceptors, in combination with expressed GT/GH candidates, may significantly accelerate the identification and characterization of further GTs/GHs responsible for plant glycosylation/modulation in the future. To enable rapid progress in this area, intensive research on the chemical and/or enzymatic synthesis of oligosaccharide acceptors and sugar nucleotide donors as well as on high-yielding production of active GT candidates in different expression systems is required. In this direction, a JBEI (The Joint BioEnergy Institute) GT Collection with almost 500 GTs from *Arabidopsis* and rice were cloned in-frame into Gateway technology compatible vectors to readily enable downstream applications (Lao et al., 2014). Either more collective resources from our laboratories (a major barrier for individual groups when research funding is scarce) or commercial intervention (which would require the same importance placed on plant biology as medical research where such resources are provided) are necessary to drive functional genomic approaches in plant glycobiology.
• *Structural diversity in N-* and *O-glycans present in plant glycoproteins*. Although some progress has been made recently, the precise *N*-glycan composition of individual native plant glycoproteins from different cells or tissues is only partially known (Xu et al., 2016; Zeng et al., 2018). Future efforts will aim to obtain a more comprehensive picture on *N*-glycan composition within specific glycoproteins to identify distinct *N*-glycan structures that are causative for a specific phenotype. In the same vein, determining functional roles for individual HRGP *O*-glycoproteins has been hampered by our failure to directly characterize each of these complex *O*-glycan structures. Only few studies have been able to purify AGPs and analyze their glycan structural variations in detail (Tan et al., 2004; Tryfona et al., 2010, 2012, 2014; Pfeifer et al., 2020). Biochemical characterization needs to be linked to detailed functional studies (e.g., site-directed mutagenesis). In general, functional validation is experimentally much more complex as well as time-consuming compared to the biochemical quantitation of the *O*-glycosylation levels. Furthermore, small changes in *O*-glycosylation in AGPs/FLAs and in EXTs can result in either activation or inactivation of their *in vivo* functions and can have an effect on their subcellular localization targeting (Velasquez et al., 2015b; Xue et al., 2017; Borassi et al., 2020; Seifert, 2020), so the functional relevance of each event cannot directly be inferred from large-scale quantitative analysis. A dual convergent approach between both enzymology/biochemistry and genetics is required to address this important aspect of plant glycoprotein structural diversity at the single cell level.
• *Overcome functional genetic redundancy of plant glycoproteins*. Addressing genetic redundancy and functional overlap might be achieved by using multiplex CRISPR-CAS9/genome editing/gene knock-out technology. Some recent reports have used this approach to overcome functional redundancy in AGPs (Moreira et al., 2020) and in GTs (e.g., GLCATs) acting on AGPs (Zhang et al., 2020). This might be extended to investigate their function in other plant species.
• *Plant glycoproteome-interactome.* Finding new proteins associated with plant glycoproteins, plant GTs and GHs will expand our knowledge on the regulatory aspects of plant glycobiology. New techniques such as proximity labeling (e.g., APEX, TurboID, etc.) together with the existing tools for detecting *in vivo* protein-protein interactions (e.g., BiFC, TriFC, FRET, etc.) will allow us to improve our plant glycobiology interactome inventory. Deeper integration of the *N*-and *O*-glycosylation pathway into the broader context of plant cell biology and systems biology is necessary. We envisage the development of a broad atlas of glycomes across plant tissues and cell types to integrate protein glycosylation features into plant gene and protein databases.

## Author Contributions

KJ, MD, CR, and FP analyzed the references, wrote the manuscript, and helped on the figures design. RS, GS, AB, and JE analyzed the references, supervised the project, and wrote the manuscript. All authors have read the manuscript and have approved this submission.

## Conflict of Interest

The authors declare that the research was conducted in the absence of any commercial or financial relationships that could be construed as a potential conflict of interest.

## Footnotes

1 www.cazy.org/
